# A study on bile from patients with recurrent common bile duct stones and cholangiocarcinoma following ERCP using non-targeted metabolomics

**DOI:** 10.3389/fmed.2026.1770095

**Published:** 2026-04-16

**Authors:** Yong-Kai Kuang, Yu-Tong Wu, Dan-Dan Wang, Gao-Feng Lu

**Affiliations:** Department of Gastroenterology, Second Affiliated Hospital of Zhengzhou University, Zhengzhou, Henan Province, China

**Keywords:** bile, cholangiocarcinoma, common bile duct stones, non-targeted metabolomics, recurrence

## Abstract

**Objective:**

This study aimed to analyze bile samples from patients with common bile duct stones and cholangiocarcinoma using non-targeted liquid chromatography-mass spectrometry (LC-MS)-based metabolomics, in order to identify disease-associated differential metabolites and metabolic pathways, and to establish metabolite-based models for predicting bile duct stone recurrence and facilitating diagnosis of cholangiocarcinoma.

**Methods:**

Bile samples and clinical data were collected from 50 patients who underwent ERCP with nasobiliary drainage tube placement at the Second Affiliated Hospital of Zhengzhou University between January and June 2025. Among them, 20 cases were primary choledocholithiasis (PDS), 15 cases were recurrent choledocholithiasis (RDS) (with ≥6 months since previous choledocholithiasis detection), and 15 with cholangiocarcinoma (CCA). LC-MS was used to detect small-molecule metabolites in bile. Principal Component Analysis (PCA) and Orthogonal Partial Least Squares Discriminant Analysis (OPLS-DA) were performed on the results. Differential metabolites were identified using metabolomics databases. The screening criteria were set at *p* < 0.05 and fold change <0.67 or >1.50, combined with variable importance projection >1.00. Pathway enrichment analysis was performed. Based on the selected differential metabolites, predictive models for common bile duct stone recurrence and cholangiocarcinoma were established, and preliminary model evaluation was conducted using receiver operating characteristic (ROC) curves.

**Results:**

LC-MS identified a total of 174 metabolites with secondary match names. Based on screening criteria, 32 differentially expressed metabolites were selected, including 17 between the PDS and RDS groups, 8 between the PDS and CCA groups, and 7 between the RDS and CCA groups. Significant enrichment was observed in the unsaturated fatty acid, amino acid, and sphingolipid metabolic pathways. Among the 17 differential metabolites in the PDS and RDS groups, four metabolites with consistent trend changes and AUC values exceeding 0.7 were selected to construct a common bile duct stone recurrence model. The area under the ROC curve reached 0.92, with a sensitivity of 86.7% and a specificity of 90.2%. Additionally, in both the stone group and cholangiocarcinoma group, the diagnostic efficacy of the novel tumor marker combination of arachidonic acid and dimethyl phosphate surpassed that of the traditional tumor marker combination of CA19-9 and CEA.

**Conclusion:**

Recurrent common bile duct stones and cholangiocarcinoma patients exhibit distinct alterations in bile metabolic profiles. Specific combinations of bile metabolites hold promise as potential biomarkers for diagnosing recurrent common bile duct stones and cholangiocarcinoma.

## 1 Introduction

Gallstone disease is characterized by the formation of calculi within the gallbladder or biliary tree. Depending on their anatomical location, gallstones are classified as cholecystolithiasis or intrahepatic and extrahepatic choledocholithiasis. Globally, 10–15% of adults are affected by gallstone disease, and common bile duct stones (CBDS) account for up to 30% of these cases (1, 2). With the progressive refinement of endoscopic retrograde cholangiopancreatography (ERCP) techniques, the initial stone clearance rate has improved to approximately 70.9–97.3%. Nevertheless, stone recurrence remains common, occurring in 9.8–25% of patients (3–6).

Persistent stone recurrence and long-term inflammatory stimulation significantly increase the risk of cholangiocarcinoma, with the 10-year incidence reported to reach up to 2.64% (7). As a highly invasive malignant tumor, cholangiocarcinoma shows a continuously rising global mortality trend. In China, its incidence increased by 202.86% from 1991 to 2021 (8). Despite this growing burden, there is currently a lack of effective clinical biomarkers for accurately predicting choledocholithiasis recurrence, and early diagnosis of cholangiocarcinoma remains challenging.

Common tumor markers such as carcinoembryonic antigen (CEA) and carbohydrate antigen 19-9 (CA19-9), exhibit limited specificity and are unreliable for distinguising biliary tract inflammation from cholangiocarcinoma or other malignancies. Consequently, identifying novel and reliable biomarkers is an urgent clinical need. Traditionally, urine has been used to study urological diseases (9), sweat for dermatological conditions (10), and cerebrospinal fluid for neurological disorders (11). Bile, which originates directly from the biliary tract, may more accurately reflect local pathophysiological changes in biliary diseases. Moreover, the biliary tract harbors a unique microbiome, whose dysregulation plays a critical role in the formation and recurrence of choledocholithiasis, as well as in the development of cholangiocarcinoma (12, 13).

Non-targeted metabolomics enables comprehensive and unbiased profiling of small-molecule metabolites within biological systems and is therefore well suited for discovering novel metabolic signatures and potential biomarkers. Recent studies have identified distinct bile microbiome compositions and metabolic profiles in patients with choledocholithiasis and cholangiocarcinoma (14). However, important gaps remain, including unclear metabolic differences between primary and recurrent choledocholithiasis and a lack of bile metabolite signatures capable of predicting disease recurrence or facilitating cholangiocarcinoma diagnosis. Therefore, the present study employs non-targeted metabolomics to systematically analyze bile metabolite profiles among patients with primary and recurrent common bile duct stones and those with cholangiocarcinoma, aiming to identify core differential metabolites, explore altered metabolic pathways, and construct metabolite-based predictive models.

## 2 Subjects and methods

### 2.1 Ethics approval and consent to participate

This study adheres to the Declaration of Helsinki and has been approved by the Ethics Committee of the Second Affiliated Hospital of Zhengzhou University (Approval Number: KY2025568).

### 2.2 Study population

Patients diagnosed with common bile duct stones or cholangiocarcinoma who underwent ERCP treatment at the Second Affiliated Hospital of Zhengzhou University between January 2025 and June 2025. This study was approved by the Ethics Committee of the Second Affiliated Hospital of Zhengzhou University (Approval Number: KY2025568).

### 2.3 Inclusion and exclusion criteria

*Inclusion criteria:* (1) Patients or their families consent to participate in this study and sign an informed consent form; (2) Patients with preoperatively confirmed common bile duct stones or cholangiocarcinoma via imaging or pathology who undergo nasobiliary drainage placement; (3) Patients with recurrent common bile duct stones were required to have had their most recent episode of choledocholithiasis detected at least 6 months earlier; (4) No intrahepatic bile duct stones or other malignant tumors. *Exclusion criteria:* (1) Patients with concomitant metabolic or autoimmune disorders, such as hyperthyroidism, hypothyroidism, diabetes mellitus, hyperlipidemia, gout, or rheumatoid arthritis; (2) Recent use of corticosteroids, lithotriptic agents, or other medications significantly affecting metabolism; (3) History of surgical stone removal or prior radiotherapy/chemotherapy.

### 2.4 Grouping and sample collection

Based on inclusion and exclusion criteria, 20 patients with primary common bile duct stones (PCS group), 15 patients with recurrent common bile duct stones (RCS group), and 15 patients with cholangiocarcinoma (CCA group) were selected. The specific sample collection procedure is as follows: (1) After an 8-h postoperative fasting period, the patient was placed in the supine position; (2) Under sterile conditions, 5 mL of bile was collected; (3) Samples were centrifuged at 4 °C (3,000 × g for 15 min) within 30 min to remove precipitates and cellular debris; (4) Aliquots of 500 μL were transferred to Eppendorf tubes, rapidly frozen in liquid nitrogen, and stored at −80 °C.

### 2.5 Major instruments, equipment, and reagents

High-performance liquid chromatography system (Agilent 1260 Infinity HPLC, USA); Orbitrap mass spectrometer (Thermo Scientific, USA); Chromatography column (Waters ACQUITY UPLC HSS T3, USA); High-speed refrigerated centrifuge (5810R, Germany); Vortex mixer (MIX-25P, China); −80 °C ultra-low temperature freezer (Thermo Fisher Scientific, USA); Flake ice machine (IMS-200, China); LC-MS grade formic acid, methanol, and acetonitrile were purchased from Tianjin Siyou Fine Chemical Co., Ltd., China.

### 2.6 Methods

#### 2.6.1 Statistical analysis

Clinical data were collected and analyzed using SPSS 27.0 statistical software. Results are presented as mean ± standard deviation (x̄ ± s), median, and interquartile range. Comparisons between groups were conducted using independent-samples *t*-tests. Quantitative data were analyzed using one-way analysis of variance (ANOVA), Tukey’s *post hoc* test, and Mann–Whitney U rank-sum test. Categorical data were evaluated using the chi-square (*χ*^2^) test. A *p* value < 0.05 was considered statistically significant.

#### 2.6.2 Sample processing and data acquisition

Sample preprocessing: Thaw collected bile samples on ice and vortex for 1 min to ensure complete mixing. Transfer 200 μL of each sample into an Eppendorf tube and add 800 μL of extraction solution (methanol:acetonitrile, 1:1). Vortex for 30 s and incubate at −20 °C overnight. Centrifuge at 4 °C (3,000 rpm, 15 min) and collect the supernatant for storage. Prepare pooled quality control (QC) samples by combining equal volumes of all samples for analysis.

Data acquisition: Select mobile phase A: 0.05% formic acid solution; mobile phase B: acetonitrile. Elution on a T3 column (100 mm × 2.1 mm, 1.8 μm) followed this gradient: 0–1 min, 95% A; 1–5 min, 90% A; 5–8 min, 80% A; 8–12 min, 5% A; 12–15 min, 95% A. Flow rate was 0.3 mL/min, injection volume was 5 μL, and column temperature was maintained at 40 °C. Mass spectrometry analysis was performed in both positive and negative ion modes with the following parameters: ion source temperature 300 °C, spray voltage 3.2 kV, auxiliary gas flow 15 arb, scan range 50–1,200 m/z, and scan time 50 ms.

#### 2.6.3 Raw data preprocessing

Raw LC-MS data were processed using the XCMS Online platform. Key parameters were set as follows: chromatographic peak width of 5–30 s, mass accuracy tolerance of 0.015 Da, and signal-to-noise ratio threshold of 10. The software automatically detected and integrated metabolite features across all samples, generating a two-dimensional data matrix containing retention time, mass-to-charge ratio (m/z), and peak intensity for each detected feature.

#### 2.6.4 Multivariate statistical analysis

To characterize global metabolic differences among groups, multivariate statistical analyses were performed on the preprocessed data. Unsupervised principal component analysis (PCA) was first conducted using SIMCA version 14.1 (Umetrics, Sweden) to visualize overall sample distribution, clustering patterns, and potential outliers. Subsequently, supervised orthogonal partial least squares discriminant analysis (OPLS-DA) was applied to enhance group separation and identify metabolites contributing to intergroup differences by separating predictive variation from orthogonal (non-correlated) variation.

Models were built using the first two principal components and validated by seven-fold cross-validation. Model performance was evaluated using *R*^2^*X* and *R*^2^*Y* values, representing the explained variance of predictors and response variables, respectively, and *Q*^2^ values indicating predictive capability. To assess potential overfitting, permutation tests with 200 iterations were performed by randomly permuting class labels and comparing the resulting *Q*^2^ values with those of the original model.

#### 2.6.5 Differential metabolite screening and identification

Differential metabolites were screened based on the following combined criteria: (1) variable importance in projection (VIP) score >1.0; (2) fold change (FC) ≥ 1.2 or ≤0.8 between groups; (3) statistical significance with *p* < 0.05. Metabolites meeting all three criteria were considered significant. Structural identification of differential metabolites was achieved by matching accurate mass measurements and MS/MS fragmentation patterns against public databases, including the Human Metabolome Database (HMDB), METLIN, MassBank, LipidMaps, and mzCloud.

#### 2.6.6 Metabolic pathway enrichment and clustering analysis

Identified differential metabolites were imported into MetaboAnalyst 5.0 for pathway enrichment analysis based on the Kyoto Encyclopedia of Genes and Genomes (KEGG) database. Enrichment results were visualized using bubble plots, where each bubble represents a metabolic pathway, with the x-axis indicating pathway impact derived from topology analysis and the y-axis representing enrichment significance. Pathways with *p* < 0.05 were considered significantly associated with the studied phenotypes. Hierarchical clustering analysis was performed using MetaboAnalyst 5.0 to generate heatmaps illustrating relative abundance patterns of differential metabolites in bile samples across the PCS, RCS, and CCA groups.

#### 2.6.7 Receiver operating characteristic curve analysis

Receiver operating characteristic (ROC) curve analysis was conducted to evaluate the diagnostic performance of differential metabolites. The area under the ROC curve (AUC) was calculated for each metabolite, and an AUC ≥ 0.80 was considered indicative of good diagnostic accuracy.

#### 2.6.8 Construction and evaluation of predictive and diagnostic models

Based on the identified differential metabolites, predictive models for choledocholithiasis recurrence and diagnostic models for cholangiocarcinoma were constructed. Metabolites with consistent intergroup trends and high AUC values were selected as candidate predictors. Multivariate logistic regression analysis was applied to develop combined models incorporating multiple metabolites. Internal validation was performed using cross-validation to reduce overfitting and improve model robustness. Model performance was assessed by ROC curve analysis, with calculation of AUC, sensitivity, and specificity.

## 3 Results

### 3.1 General information

A comparison of clinical characteristics between the two groups showed no statistically significant differences in age, sex, or body mass index. However, significant differences were observed in total bilirubin, direct bilirubin, alkaline phosphatase, *γ*-glutamyltransferase, alanine aminotransferase, aspartate aminotransferase, carcinoembryonic antigen, and carbohydrate antigen 19-9 (*p* < 0.05). Among these, total bilirubin, alanine aminotransferase, and carcinoembryonic antigen demonstrated highly significant differences (*p* < 0.01). The detailed results are presented in Table 1.

**Table 1:** General characteristics of participants.

Project	Stone group	Bile duct cancer group	*X*^2^/*F*/*Z*	*p*-value
Total	35	15	/	/
Male/female	14/21	8/7	0.552	0.821
Age	55.72 ± 17.82	61.32 ± 10.54	1.464	0.22
BMI	23.2 ± 3.9	20.1 ± 3.1	2.432	0.15
TBil	59.54 (19.22, 143.25)	186.84 (65.56, 355.66)	25.574	<0.01
DBil	45.56 (12.41, 122.12)	125.26 (45.23, 231.56)	12.323	<0.05
ALP	157.56 (68.86, 311.25)	211.33 (178.12, 403.01)	10.554	<0.05
GGT	176.30 (82.21, 389.11)	283.22 (181.55, 493.75)	8.354	<0.05
ALT	67.50 (43.53, 125.85)	92.00 (53.21, 104.44)	16.575	<0.01
AST	45.00 (28.50, 85.50)	69.50 (42.50, 98.75)	11.213	<0.05
CEA	1.30 (0.75, 2.21)	6.56 (2.34, 35.58)	15.329	<0.01
CA19-9	35.26 (15.21, 142.43)	95.63 (21.02, 423.43)	5.211	<0.05

(1) BMI: body mass index; TBil: total bilirubin; DBil: direct bilirubin; ALP: alkaline phosphatase; GGT: gamma-glutamyl transpeptidase; ALT: alanine transaminase; AST: aspartate transaminase; CEA: carcinoembryonic antigen; CA19-9: carbohydrate antigen 19-9; (2) Differences were compared using chi-square tests for gender, one-way ANOVA for age, and Mann–Whitney U tests for other blood parameters.

### 3.2 Principal component analysis

**Figure 1:**
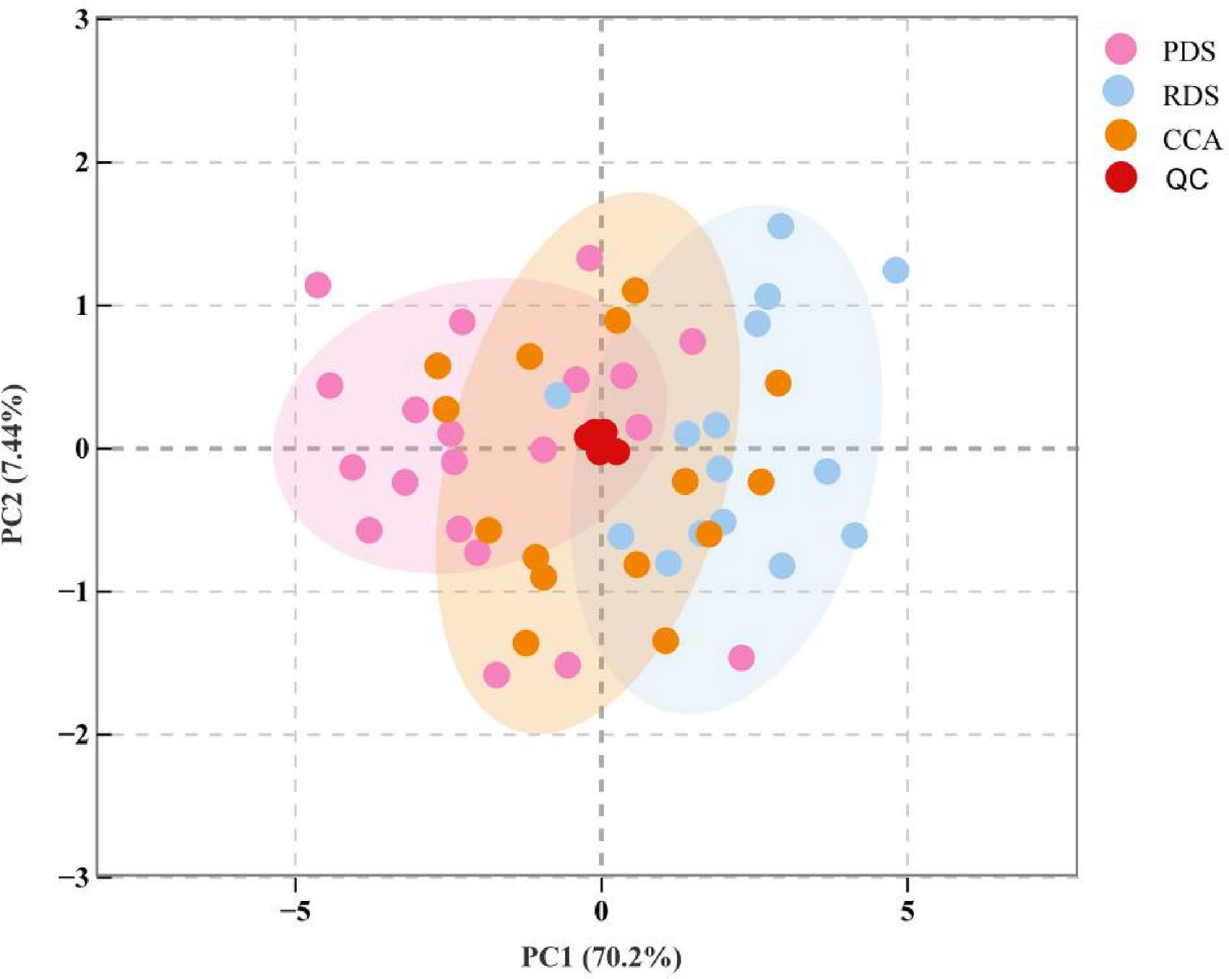
PCA score plot of three sample groups. PDS group represents patients with first-time common bile duct stones; RDS denotes recurrent common bile duct stones; CCA indicates cholangiocarcinoma patients; QC denotes quality control samples. Each dot represents one sample, with ellipses denoting 95% confidence intervals.

Three principal component analysis (PCA) score plots are shown in Figure 1. QC samples exhibit no significant deviation, indicating high sample reliability. Instrument stability and reproducibility during bile metabolomics measurement were excellent. Samples demonstrate a clear separation trend along the PC1 axis, revealing distinct differences in small molecules within bile samples. Although partial overlap exists between PDS and RDS, the separation trend is evident, with the recurrent group’s distribution clustering closer to the cholangiocarcinoma group.

### 3.3 Orthogonal partial least squares discriminant analysis

**Figure 2:**
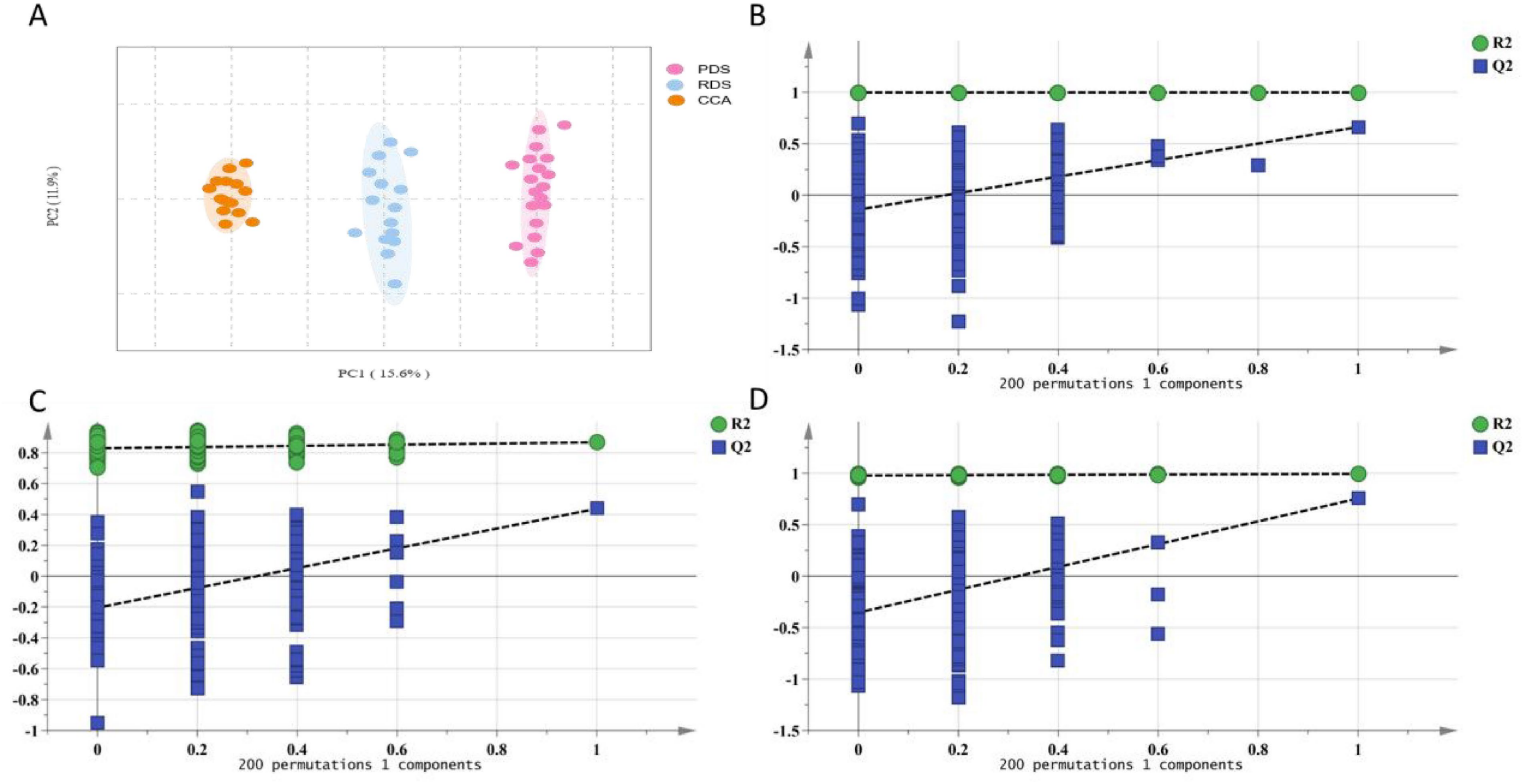
OPLS-DA and permutation test plot. **(A)** OPLS-DA scatter plot score; **(B)** Permutation test plot for PDS-RDS; **(C)** Permutation test plot for PDS-CCA; **(D)** Permutation test plot for RDS-CCA.

Notably, samples in the recurrence group demonstrated tighter clustering, indicating greater homogeneity in their metabolomic profiles. In contrast, the cholangiocarcinoma group exhibited broader dispersion, reflecting higher inter-individual variability. The partial overlap among the three groups suggests shared metabolic features; however, distinct group-specific patterns are also evident and warrant further investigation. Building upon this overall separation trend, Orthogonal Partial Least Squares Discriminant Analysis (OPLS-DA) was subsequently applied. The results revealed significant differences in bile metabolomes, with model parameters shown in Table 2, demonstrating robustness and predictive capability. Subsequently, a 200-iteration permutation test was performed. The *R*^2^ and *Q*^2^ values for the PDS vs. RDS, RDS vs. CCA, and PDS vs. CCA groups were (0.999 and −0.322; 0.921 and −0.425; 0.977 and −0.354), with *p*-values below 0.01, effectively ruling out overfitting (see Figure 2).

**Table 2:** OPLS-DA model parameters.

Group	*R* ^2^ *X*	*R* ^2^ *Y*	*Q* ^2^
PDS-RDS	0.577	0.999	0.662
PDS-CCA	0.509	0.867	0.537
RDS-CCA	0.615	0.995	0.754

*R*^2^*X* denotes the model’s explanatory power for categorical variable *X*; *R*^2^*Y* denotes the model’s explanatory power for categorical variable *Y*; *Q*^2^ denotes the model’s predictive capability.

### 3.4 Screening, identification, and pathway analysis of differential metabolites

**Figure 3:**
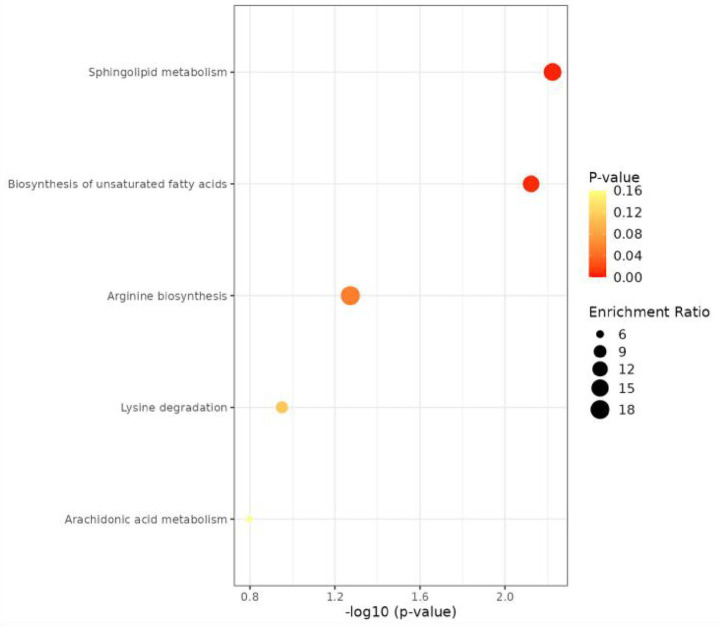
Enrichment bubble plot of metabolic pathways for differentially expressed metabolites. Dot size represents the percentage of differentially metabolized metabolites in the metabolic pathway.

Among the 3,568 identified metabolites, 174 with secondary match names (MS2) were further screened via databases. Using *p* < 0.05, fold change <0.67 or >1.50, combined with a variable importance projection (VIP) > 1.00. From these, 17 differential metabolites with AUC values >0.7 were selected: 11 from PDS vs. RDS, 3 from PDS vs. CCA, and 3 from RDS vs. CCA (Table 3). Pathway enrichment analysis of the differential metabolites revealed predominant enrichment in the unsaturated fatty acid, amino acid, and sphingolipid metabolism pathways, as shown in Figure 3.

**Table 3:** Differentially metabolized compounds among three sample groups.

Group	Differential metabolites	RT (min)	VIP	*p*-value	Change multiple
PSD-RSD	1-tetradecylamine	7.79	1.70	0.0009	0.761
	Arachidonic acid	12.68	1.80	0.0341	2.517
	Docosahexaenoic acid	12.51	1.78	0.0169	0.342
	Hippuric acid	3.96	1.02	0.0337	20.839
	L-carnitine	0.86	3.57	0.0126	4.526
	N, N-dimethylaniline	13.69	3.94	0.0024	0.442
	3-indolepropionic acid	3.53	7.26	0.0235	5.266
	Phytosphingosine	7.44	1.22	0.0019	0.776
	Proline betaine	6.25	2.15	0.0258	6.259
	L-citrulline	4.33	2.37	0.0326	0.445
	Lithocholic acid	6.36	1.99	0.0441	0.259
PSD-CCA	2-aminooctadec-4-yne-1,3-diol	8.02	1.22	0.0259	2.391
	Ritalinic acid	3.35	1.32	0.0277	3.768
	Sphingosine	8.59	2.58	0.0230	2.238
RSD-CCA	Methyl palmitate	13.14	3.91	0.0110	8.213
	Dimethyl phosphate	9.019	2.88	0.0168	3.239
	SM(d18:1/16:0)	12.81	3.63	0.0329	4.021

RT: retention time; VIP: variable importance projection.

### 3.5 Hierarchical clustering analysis of differentially expressed metabolites

**Figure 4:**
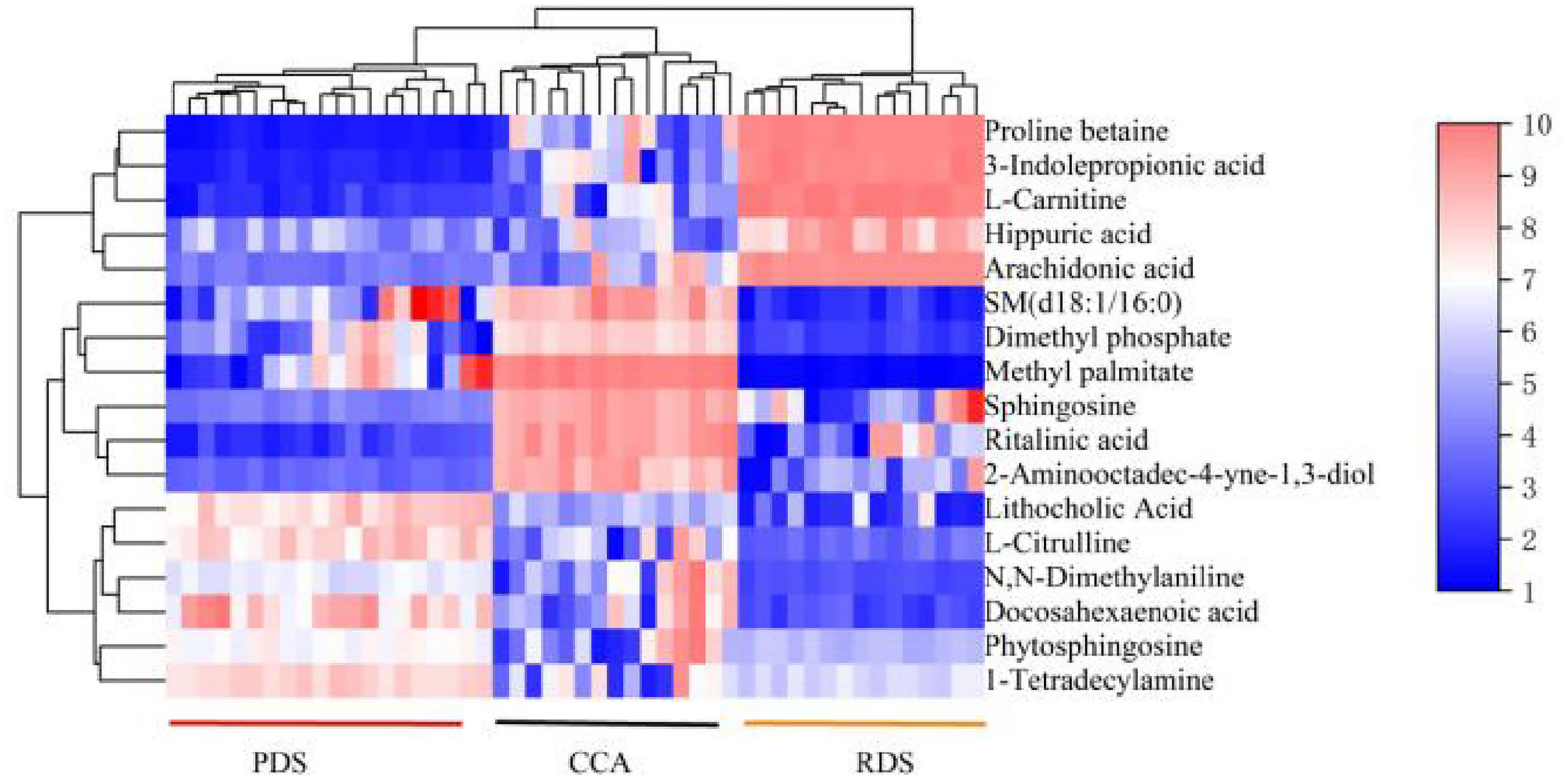
Hierarchical clustering heatmap of differentially metabolized compounds f. Each row in the figure represents a differentially expressed metabolite, and each column represents a sample.

Hierarchical clustering analysis is an unsupervised expression visualization technique that constructs heatmaps reflecting metabolic similarity among samples. Hierarchical clustering of the 17 selected differentially expressed metabolites clearly revealed distinct metabolic profiles across disease groups, as shown in Figure 4. All samples were distinctly grouped into three major clusters based on their metabolite expression profiles, which highly aligned with clinical diagnostic classifications (PDS, RDS, and CCA). Specifically, 11 differentially expressed metabolites between the PDS and RDS groups (e.g., arachidonic acid, proline betaine) exhibited a regular expression gradient, showing an overall upregulation trend in the RDS group. This effectively distinguished recurrent patients from initial patients in the clustering. Concurrently, cholangiocarcinoma (CCA) samples exhibited a distinct metabolic pattern. Their characteristic differential metabolites (e.g., 2-aminooctan-4-yne-1,3-diol, sphingosine) formed a signature expression profile distinct from the stone group, enabling CCA samples to cluster independently. This visualization analysis provides intuitive confirmation of the significant heterogeneity in bile metabolomics across different disease states, offering robust support for subsequent diagnostic model development.

### 3.6 Development of predictive models for the recurrence of common bile duct stones and the occurrence of cholangiocarcinoma

**Figure 5:**
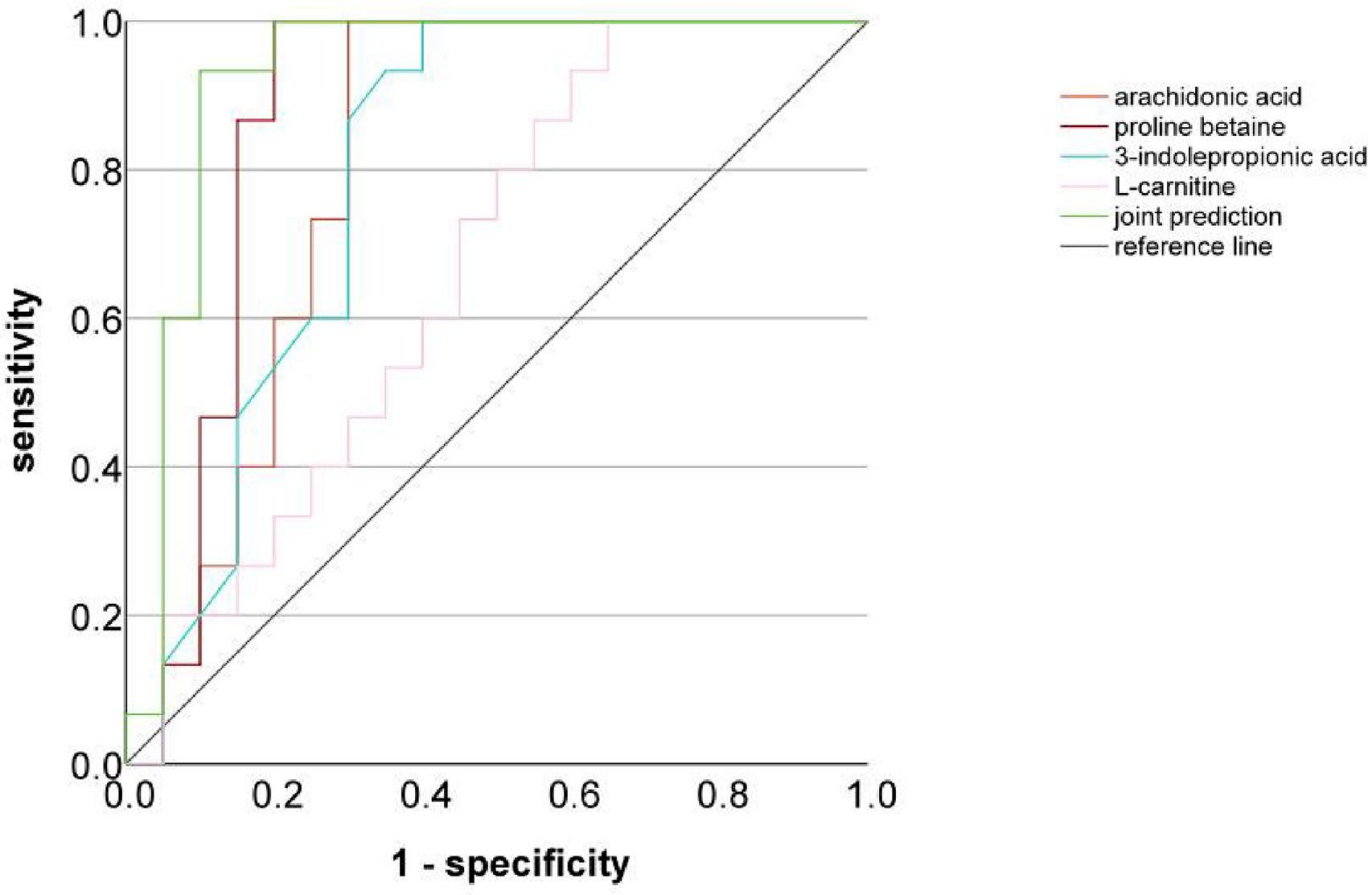
ROC curve analysis of differential metabolites between the PDS and RDS groups.

**Figure 6:**
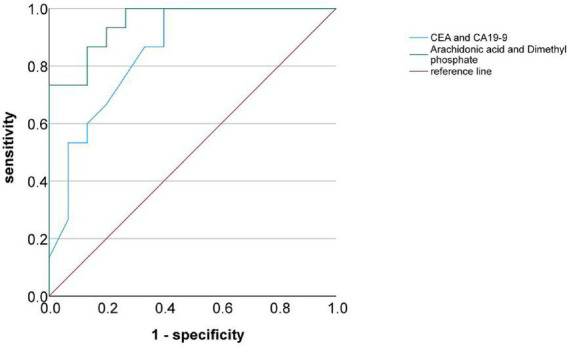
ROC curve analysis of differential metabolites and conventional tumor markers.

Based on the significant results from the screening of differential metabolites and hierarchical cluster analysis, we further constructed a predictive model for common bile duct stone recurrence and cholangiocarcinoma development. Among the 11 differential metabolites identified between the PDS and RDS groups, arachidonic acid,3-Indolepropionic acid, Proline betaine, and L-carnitine—metabolites that exhibited consistent trends and relatively high AUC values—were selected to construct a predictive model for the recurrence of common bile duct stones. The model yielded an AUC of 0.92, with a sensitivity of 86.7% and a specificity of 90.2%, indicating strong diagnostic performance (Figure 5). In both the PDS versus CCA and the RDS versus CCA comparisons, arachidonic acid and dimethyl phosphate—metabolites with relatively high AUC values—were selected to construct a predictive model for cholangiocarcinoma. This model achieved an AUC of 0.94, with a sensitivity of 86.2% and a specificity of 89.4%, demonstrating superior predictive performance compared with the conventional tumor markers CA125 and CA19-9 (Figure 6).

## 4 Discussion

This study analyzed bile metabolic profiles obtained after ERCP from patients with primary and recurrent common bile duct stones and cholangiocarcinoma using non-targeted metabolomics. Distinct metabolic alterations were identified during stone recurrence and malignant transformation, providing insights into disease mechanisms and a basis for biomarker discovery. Pathway enrichment analysis revealed that differential metabolites between the PDS and RDS groups were mainly involved in unsaturated fatty acid and amino acid metabolism, whereas differences between the stone groups and cholangiocarcinoma were predominantly associated with sphingolipid metabolism. These metabolic disturbances not only reflect disease progression but also form the biological foundation for the metabolite-based prediction and diagnostic models developed in this study. Although liver function indices can reflect biliary obstruction, they often lack specificity. The metabolites identified in this study provide deeper biological insights—such as information on inflammatory mediators and metabolic pathways—that cannot be captured by conventional biochemical markers.

### 4.1 Metabolic pathways of unsaturated fatty acids

In this study, the unsaturated fatty acid metabolic pathway was identified as significantly altered between the PDS and RDS groups, suggesting that dysregulated lipid metabolism plays a critical role in the development of common bile duct stones and their potential progression to cholangiocarcinoma. Unsaturated fatty acids not only constitute a major component of bile but their metabolites are also extensively involved in inflammation, immune regulation, and cellular signaling processes (15–17).

From a pathophysiological perspective, unsaturated fatty acids directly influence the solubility and stability of bile by modulating the ratio of cholesterol, bile acids, and phospholipids (18). The significant alterations observed in fatty acid metabolism in the stone groups (PDS and RDS) indicate that imbalances in bile composition may persist throughout stone formation and recurrence. Previous studies have shown that omega-6 fatty acid metabolism generates pro-inflammatory mediators such as prostaglandins and leukotrienes (19), which can inhibit biliary smooth muscle contraction and prolong bile retention in the ductal system, thereby facilitating cholesterol precipitation and crystal formation (20). Chronic inflammation mediated by unsaturated fatty acids has also been proposed to act as a bridge linking long-term biliary irritation and epithelial injury, providing a microenvironment conducive to malignant transformation. Furthermore, unsaturated fatty acids can modulate gut microbiota composition (21), leading to excessive conversion of bile acids into secondary bile acids such as deoxycholic acid, which have lower cholesterol solubility (22, 23). Upon returning to the liver, these secondary bile acids increase the lithogenicity of bile and exert cytotoxic effects on biliary epithelium, resulting in epithelial damage. Taken together with the dysregulated unsaturated fatty acid metabolism observed in this study, it is plausible that persistent lipid metabolic disturbances contribute to the progression from common bile duct stones to cholangiocarcinoma through a pathway of “enhanced bile lithogenicity–chronic biliary injury–sustained inflammatory activation.”

### 4.2 Amino acid metabolic pathways

Multiple amino acid–related metabolites exhibited significant differences among the PDS, RDS, and CCA groups, and amino acid metabolism pathways were highly enriched in pathway analysis. These findings suggest that dysregulated amino acid metabolism is not merely a bystander phenomenon associated with stone formation but may actively contribute to the initiation and progression of biliary pathology. Amino acids do not act as passive organic constituents in gallstone formation; rather, they influence bile homeostasis and biliary function through multiple mechanisms. Bile acids must conjugate with glycine or taurine in the liver before being secreted into bile. The significant alterations in amino acid metabolic pathways observed in this study suggest that the generation of conjugated bile acids may be affected, potentially reducing bile stability and promoting cholesterol precipitation (24). In terms of inflammation and biliary dynamics, abnormal arginine metabolism can affect nitric oxide production, thereby disrupting biliary contractility and bile flow and exacerbating bile stasis (25). Chronic bile stasis and inflammation are well-established risk factors for cholangiocarcinoma, consistent with our observation of significant differences in amino acid metabolism between the stone groups and the CCA group. Tryptophan metabolism was not significantly altered among the three groups in this study; however, previous studies have shown that tryptophan can be metabolized by gut microbiota into indole derivatives, which act as ligands for the aryl hydrocarbon receptor and exert anti-inflammatory and protective effects in cholangiocytes. Insufficient tryptophan metabolism may therefore weaken this protective mechanism (26). Taken together, dysregulated amino acid metabolism may influence bile composition, biliary motility, and the inflammatory microenvironment, thereby playing a multifaceted role in the formation, recurrence, and malignant transformation of common bile duct stones.

### 4.3 Sphingolipid metabolic pathway

In our study, sphingosine was detected in both the RDS and CCA groups, and sphingolipid metabolism emerged as a significantly altered pathway. We speculate that sphingosine may serve as a key mediator linking the recurrence of bile duct stones to cholangiocarcinoma development. Sphingolipids, composed of a sphingosine backbone and fatty acids, are structurally complex phospholipids that not only constitute cellular membranes but also act as critical signaling molecules. Key metabolites within sphingolipid pathways, such as ceramide, sphingosine-1-phosphate (S1P), and glucosylceramide, play central roles in regulating cell proliferation, apoptosis, migration, invasion, and inflammatory responses. The core of sphingolipid metabolism lies in the balance between ceramide-induced apoptosis and S1P-mediated survival. In tumor biology, the ratio of these two metabolites has been aptly termed the “sphingolipid rheostat,” which determines whether a cell undergoes death (high ceramide levels) or proliferation (high S1P levels) (27). S1P can also stimulate angiogenesis, supplying nutrients to support tumor growth, and enhance cancer cell motility, thereby promoting distal invasion and metastasis (28). In this study, the significant differences in sphingosine-related metabolites between the stone groups and the cancer group suggest that this balance may be remodeled during disease progression.

### 4.4 Clinical interpretation and performance of metabolite-based models

A major contribution of this study is the development of metabolite-based models for predicting common bile duct stone recurrence and diagnosing cholangiocarcinoma. The recurrence prediction model achieved an AUC of 0.92 with high sensitivity and specificity, indicating strong discriminative ability. Clinically, such performance suggests potential utility in postoperative risk stratification, enabling identification of patients at high risk for recurrence who may benefit from closer follow-up or preventive interventions. The diagnostic model for cholangiocarcinoma also demonstrated favorable performance, highlighting the advantage of integrating multiple bile-derived metabolic signals rather than relying on single biomarkers (29).

### 4.5 Comparison with existing biomarkers and clinical utility

Conventional tumor markers such as CA19-9 and CEA are widely used in clinical practice but often lack specificity in the context of biliary inflammation, leading to false-positive results and limited diagnostic value for early-stage cholangiocarcinoma (30). In contrast, the metabolite combinations identified in this study, particularly those involving arachidonic acid–related metabolic networks, exhibited superior diagnostic performance. These findings suggest that bile-based metabolite panels may complement existing biomarkers and improve diagnostic accuracy in distinguishing malignant transformation from benign biliary disease, especially in patients undergoing ERCP (31).

### 4.6 Limitations and future perspectives

Several limitations of this study should be acknowledged. First, the sample size was relatively small and derived from a single center, which may limit the generalizability of the findings; multicenter studies are therefore needed to further establish the robustness and applicability of these results. Second, because obtaining bile samples from healthy individuals is clinically challenging, we included patients with first-onset choledocholithiasis as a surrogate control group to approximate the bile composition of healthy individuals as closely as possible. Third, bile sampling is inherently invasive and is not suitable for population-wide screening; however, it remains feasible in selected high-risk patients undergoing ERCP. Fourth, the model constructed based on bile metabolite profiles may serve as an adjunctive tool for the diagnosis of cholangiocarcinoma, but it currently lacks the ability to discriminate tumor stages or detect early-stage lesions. Future studies incorporating clinical staging information may further evaluate the potential utility of this model in early detection.

## 5 Conclusion

In summary, this study integrates bile metabolomics with predictive modeling to characterize metabolic alterations associated with common bile duct stone recurrence and cholangiocarcinoma. By identifying key differential metabolites and constructing metabolite-based models with favorable diagnostic performance, our findings highlight the potential clinical value of bile-derived metabolic signatures for disease risk assessment and early diagnosis, while also providing mechanistic insights into the metabolic pathways underlying disease progression.

## Data Availability

The datasets generated for this study involve human participants and are not publicly available due to privacy restrictions. Requests to access the datasets should be directed to the corresponding author.

## Glossary

AHR: aromatic hydrocarbon receptor
ALP: alkaline phosphatase
ALT: alanine transaminase
AST: aspartate transaminase
AUC: area under curve
BMI: body mass index
CA19-9: carbohydrate antigen 19-9
CBDS: common bile duct stones
CCA: cholangiocarcinoma
CEA: carcinoembryonic antigen
DBiL: direct bilirubin
ERCP: endoscopic retrograde cholangiopancreatography
GGT: gamma-glutamyl transpeptidase
LC-MS: liquid chromatography-mass spectrometry
MMC: migrating motor complex
NETs: neutrophil extracellular traps
OPLS-DA: orthogonal partial least squares discriminant analysis
PAD4: peptidyl arginine deiminase 4
PCA: principal component analysis
PDS: primary choledocholithiasis
QC: quality control
RDS: recurrent choledocholithiasis
ROC: receiver operating characteristic
RT: retention time
S1P: sphingosine-1-phosphate
SphK: sphingosinekinases
TBiL: total bilirubin
VIP: variable importance in projection
